# Prognostic Significance of Modified Advanced Lung Cancer Inflammation Index (ALI) in Patients with Small Cell Lung Cancer_ Comparison with Original ALI

**DOI:** 10.1371/journal.pone.0164056

**Published:** 2016-10-05

**Authors:** Eun Young Kim, Nambeom Kim, Young Saing Kim, Ja-Young Seo, Inkeun Park, Hee Kyung Ahn, Yu Mi Jeong, Jeong Ho Kim

**Affiliations:** 1 Department of Radiology, Gachon University Gil Medical Center, Incheon, Republic of Korea; 2 Neuroscience Research Institute, Gachon University, Incheon, Republic of Korea; 3 Division of Hematology and Oncology, Department of Internal Medicine, Gachon University Gil Medical Center, Incheon, Republic of Korea; 4 Department of Laboratory Medicine, Gachon University Gil Medical Center, Incheon, Republic of Korea; University of Nebraska Medical Center, UNITED STATES

## Abstract

**Background:**

Advanced lung cancer inflammation index (ALI, body mass index [BMI] x serum albumin/neutrophil-lymphocyte ratio [NLR]) has been shown to predict overall survival (OS) in small cell lung cancer (SCLC). CT enables skeletal muscle to be quantified, whereas BMI cannot accurately reflect body composition. The purpose was to evaluate prognostic value of modified ALI (mALI) using CT-determined L3 muscle index (L3MI, muscle area at L3/height^2^) beyond original ALI.

**Methods:**

L3MIs were calculated using the CT images of 186 consecutive patients with SCLC taken at diagnosis, and mALI was defined as L3MI x serum albumin/NLR. Using chi-squared test determined maximum cut-offs for low ALI and low mALI, the prognostic values of low ALI and low mALI were tested using Kaplan-Meier method and Cox proportional hazards analysis. Finally, deviance statistics was used to test whether the goodness of fit of the prognostic model is improved by adding mALI as an extra variable.

**Results:**

Patients with low ALI (cut-off, 31.1, n = 94) had shorter OS than patients with high ALI (median, 6.8 months vs. 15.8 months; *p* < 0.001), and patients with low mALI (cut-off 67.7, n = 94) had shorter OS than patients with high mALI (median, 6.8 months vs. 16.5 months; *p* < 0.001). There was no significant difference in estimates of median survival time between low ALI and low mALI (z = 0.000, *p* = 1.000) and between high ALI and high mALI (z = 0.330, *p* = 0.740). Multivariable analysis showed that low ALI was an independent prognostic factor for shorter OS (HR, 1.67, *p* = 0.004), along with advanced age (HR, 1.49, *p* = 0.045), extensive disease (HR, 2.27, *p* < 0.001), supportive care only (HR, 7.86, *p* < 0.001), and elevated LDH (HR, 1.45, *p* = 0.037). Furthermore, goodness of fit of this prognostic model was not significantly increased by adding mALI as an extra variable (LR difference = 2.220, *p* = 0.136).

**Conclusion:**

The present study confirms mALI using CT-determined L3MI has no additional prognostic value beyond original ALI using BMI. ALI is a simple and useful prognostic indicator in SCLC.

## Introduction

Lung cancer is the leading cause of cancer-related death worldwide and small cell lung cancer (SCLC) accounts for about 15% of all cases. Although SCLC is highly responsive to initial chemotherapy and radiotherapy, it spreads much more quickly than non-small cell lung cancer and its prognosis is poor [1, 2]. Tumor staging systems are the most important predictors of overall survival (OS), and a number of clinical indicators are also related to prognosis in SCLC patients. In particular, studies indicate that clinical characteristics, such as, gender, age, smoking status, and performance status, have prognostic significance [1, 3]. A recent study described the prognostic significance of advance lung cancer inflammation index (ALI; a systemic inflammation-based index), which was calculated by multiplying body mass index (BMI, kg/m^2^) by the serum albumin/neutrophil-lymphocyte ratio (NLR). The results obtained showed a low ALI (< 19.5) was significantly associated with a poor prognosis in SCLC [4].

Quantification of skeletal muscle mass is important for determining the presence of sarcopenia, which is an important component of cancer cachexia syndrome and significant prognostic indicator in cancer patients, because skeletal muscle mass status is associated with functional impairment, increased risk of chemotherapy-related toxicities, and reduced survival [5–10]. However, although BMI is known not to reflect body composition accurately, it is used to calculate ALI. Accordingly, we undertook this study to determine whether the modified ALI (mALI) incorporating CT-determined skeletal muscle mass has more prognostic value than ALI as originally defined.

## Materials and Methods

### Patients

We retrospectively identified patients with newly diagnosed, pathologically proven SCLC that underwent a baseline chest CT scan and a PET/CT scan from January 2010 to October of 2015 using the radiology database and medical records system at Gachon University Gil Medical Center (Incheon, Korea). Heights and weights were measured and functional statuses were recorded at first visit to our oncology department. Body mass index (BMI) was defined as weight divided by height squared (kg/m^2^).

SCLC was classified as limited or extensive. Limited stage was defined as American Joint Committee on Cancer (AJCC) stages I to III, which can be safely treated by definitive radiation therapy [11]. Treatment for SCLC included active therapies, such as, chemotherapy, chemoradiotherapy (including sequential and concurrent therapy), chest radiotherapy, and supportive care only. The institutional review board of our hospital approved this retrospective study and waived the requirement for informed patient consent.

### Neutrophil to lymphocyte ratio (NLR)

At time of diagnosis, a pretreatment venous blood sample was collected after a 12-hour overnight fast. Blood cell counts were determined using the ADVIA 2120 Hematology System (Siemens AG, Eschborn, Germany) and serum albumin, lactate dehydrogenase (LDH) levels were measured using an ADVIA 2400 Chemistry System (Siemens Healthcare Diagnostics, Sacramento, CA, USA).

### CT Image analysis

Single cross-sectional area of third lumbar vertebra (L3MA) measured on PET/CT at the time of SCLC diagnosis served as a reference standard. A six-detector CT (Siemens Medical Systems, Erlangen, Germany) equipped with lutetium oxyorthosilicate crystal PET detectors was operated using the following imaging parameters; 130 kVp, 110 mAs, 2-mm pitch, 1-s tube rotation, and a slice thickness of 5 mm, which matched the slice thickness of PET images.

Quantitative assessments of muscle areas were performed using commercially available software (Terarecon 3.4.2.11, San Mateo, CA, USA) by a subspecialty-trained chest radiologist. Tissue cross-sectional areas (cm^2^) of respective tissues in slices were computed automatically by summing appropriate pixels using the CT Hounsfield unit (HU) range -29 HU to 150 HU for skeletal muscle. After applying threshold methods using the predefined HU threshold range to each slice, boundaries between different tissues were corrected manually when necessary. L3 muscle index (L3MI, cm^2^/m^2^) was defined as the cross-sectional area of muscle at the L3 level divided by height squared as is conventional for BMI.

### Statistical analysis

Descriptive statistics are reported as proportions or means with standard deviations (SDs). For categorical variables, Pearson’s chi-squared test or Fisher’s exact test were used for intergroup comparisons. Continuous variables were compared using the Student’s *t*-test or the Mann-Whitney *U* test. ALI was defined as BMI x serum albumin/NLR and mALI was defined as CT-determined L3MI x serum albumin/NLR. Maxstat, a maximal chi-squared method in open source statistical software R (R Development Core Team, Vienna, Austria, http://www.R-project.org) was used to determine optimal cut-off points for ALI and mALI, and the prognostic significances of determined low ALI and mALI values were evaluated using Kaplan-Meier. The median survival time (MST) was compared between low ALI and low mALI by using independent two-sample z-test.

Univariable and multivariable Cox proportional hazard models were used to identify prognostic factors of survival. Variables with a *p* value of < 0.15 by the log-rank test were included in the backward stepwise multivariable analysis. Goodness of fit for the final model was assessed by calculating the likelihood ratio test. The reliability of the predictors for prognosis was assessed using a bootstrap resampling technique with 1,000 samples [12]; for each sample, analysis was performed using a backward stepwise Cox proportional hazard model. If the final prognostic model variables appear in a majority (> 50%) of the bootstrap models, the original final stepwise regression model can be judged as stable. In addition, deviance statistics was used to test whether the goodness of fit of the final prognostic model is improved by adding mALI as an extra variable [13]. A *p* values of ≤ 0.05 were considered statistically significant and the analysis was performed using SPSS for Windows ver. 19.0 (SPSS Inc., Chicago, IL, USA) and R ver 3.3.1.

## Results

### Characteristics of the study population

A total of 186 consecutive patients were included in the present study (Table 1). Mean patient age was 68.6 ± 9.4 years and 156 (83.9%) were male. Of the 186 patients, 122 (65.6%) had extensive disease at presentation. Average BMI was 22.3 ± 3.6 kg/m^2^ and average L3MI was 48.6 ± 9.6 cm^2^/m^2^.

**Table 1:** Characteristics of patients with small cell lung cancer according to advanced lung inflammation index (ALI) values.

Characteristics	All (n = 186)	ALI < 31.1 (n = 94)	ALI ≥ 31.1 (n = 92)	*p* value
**Age, years**	68.8 ± 9.4	71.5 ± 9.2	66.1 ± 8.8	< 0.001^a^
≥ 65 years	127 (67.1%)	71 (75.5%)	56 (60.9%)	0.032^b^
**Male**	156 (83.9%)	82 (87.2%)	74 (80.4%)	0.207^b^
**Smoking Status**				
Current/ex-smoker	166 (89.2%)	88 (93.6%)	78 (84.8%)	0.052^b^
Never smoker	20 (10.8%)	6 (6.4%)	14 (15.2%)	
Pack year, median (range)	35 (0–171)	40 (0–171)	30 (0–120)	0.151^c^
**Stage**				
Limited disease	64 (34.4%)	23 (24.5%)	41 (44.6%)	0.004^b^
Extensive disease	122 (65.6%)	71 (75.5%)	51 (55.4%)	
**ECOG PS**				
0–1	132 (71.0%)	53 (56.4%)	79 (85.9%)	< 0.001^b^
≥ 2	54 (29.0%)	41 (43.6%)	13 (14.1%)	
**Charlson comorbidity index**				
0	59 (31.7%)	24 (25.5%)	35 (38.0%)	0.157^b^
1–2	101 (54.3%)	57 (60.6%)	44 (47.8%)	
≥ 3	26 (14.0%)	13 (13.8%)	13 (14.1%)	
**Body mass index, kg/m**^**2**^	22.3 ± 3.6	20.8 ± 3.2	23.9. ± 3.3	< 0.001^a^
**L3 muscle index, cm/m**^**2**^	48.6 ± 9.6	46.4 ± 9.3	50.9 ± 9.4	0.001^a^
**Inflammatory markers**				
WBC, 10^9^/L	7.90 (3.58–24.11)	8.71 (3.93–24.11)	7.39 (3.58–15.02)	< 0.001^c^
Neutrophil to lymphocyte ratio	2.7 (0.8–20.6)	4.28 (2.11–20.59)	1.82 (0.80–2.84)	< 0.001^c^
Albumin, g/dL	3.95 (2.50–4.90)	3.80 (2.50–4.70)	4.10 (3.30–4.90)	< 0.001^c^
**ALI**	37.07 ± 25.3	18.4 ± 7.3	56.2 ± 22.7	< 0.001^a^
**mALI**	80.0 ± 54.3	41.3 ± 17.7	119.6 ± 50.3	< 0.001^a^
**Treatment**				0.001^d^
Chemotherapy	94 (50.5%)	49 (52.1%)	45 (48.9%)	
Chemoradiotherapy	59 (31.7%)	20 (21.3%)	39 (42.4%)	
Chest radiotherapy	2 (1.1%)	1 (1.1%)	1 (1.1%)	
Supportive care only	31 (16.7%)	24 (25.5%)	7 (7.6%)	
**First-line chemotherapy regimen**				0.018^d^
Etoposide/cisplatin	96 (62.7%)	38 (55.1%)	58 (59.0%)	
Etoposide/carboplatin	22 (14.4%)	15 (21.7%)	7 (8.3%)	
Irinotecan/cisplatin	12 (7.8%)	5 (7.2%)	7 (8.3%)	
Belotecan/ifosfamide	16 (10.5%)	5 (7.2%)	11 (13.1%)	
Etoposide monotherapy	7 (4.6%)	6 (8.7%)	1 (1.2%)	
**LDH (U/L), median (range)**	531 (282–8587)	569 (294–8587)	500 (282–2289)	0.012^c^
Elevated LDH (≥ 486 U/L)	105 (56.5%)	56 (59.6%)	49 (53.3%)	0.385^b^

Abbreviations: ECOG PS, Eastern Cooperative Oncology Group performance status; CRP, C-reactive protein; mGPS, modified Glasgow Prognostic Score; LDH, lactate dehydrogenase; ALI, Advanced lung cancer inflammation index; mALI, modified ALI. Values are means ± standard deviations.

^a^Student *t*- test,

^b^Chi-squared test,

^c^Mann-Whitney *U* test,

^d^Fisher’s exact test

### Original ALI and modified ALI

For the 186 study subjects, mean ALI and mALI values were 37.07 ± 25.3 and 80.0 ± 54.3 respectively, and a significant correlation was found between ALI and mALI (r = 0.962, *p* < 0.001). Cut-offs for low ALI and low mALI as determined by the maximum chi-squared test were 31.1 and 67.7, respectively; 94 patients (50.5%) had a low ALI (< 31.1) and 94 had a low mALI (< 67.7) (Table 2), and for 172 patients (92.4%) categorizations by ALI and mALI concurred. Patients with a low ALI (n = 94) were older and had higher proportions of patients with extensive disease, poor performance status, supportive care only, and higher LDH levels than patients with a high ALI (Table 1).

**Table 2:** Numbers of patients categorized according to ALI and mALI (ALI) cut-off values.

	Low ALI (ALI < 31.1)	ALI **≥** 31.1
Low mALI (mALI < 67.7)	85	7
mALI ≥ 67.7	7	87

Abbreviations: ALI, Advanced lung cancer inflammation index; mALI, modified ALI

### Prognostic significances of ALI and mALI

**Fig 1:**
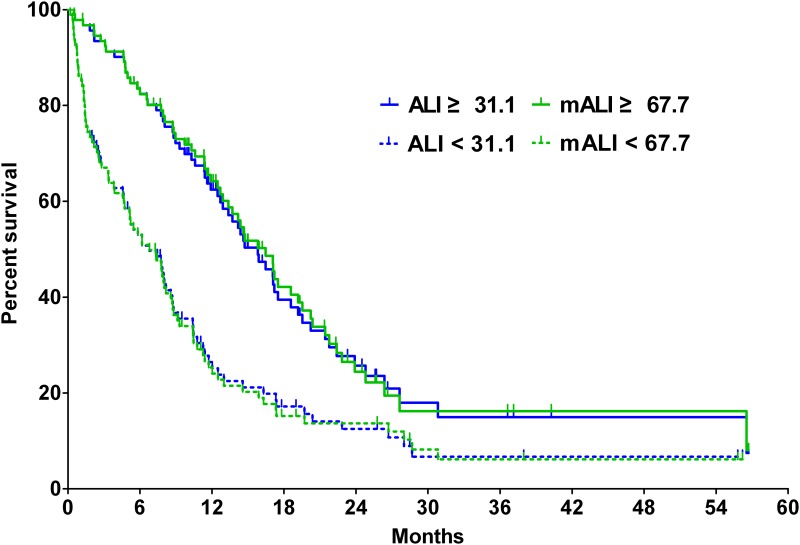
Kaplan-Meier curves of advance lung cancer inflammation index (ALI) and modified ALI (mALI).

Over a median follow-up of 29.0 months (95% confidence interval [CI], 19.7 to 38.3 months), 141 patients (83.5%) died. For all 186 study subjects, median OS was 10.6 months (95% CI, 8.6 to 12.6 months). Patients with a low ALI had shorter OS than patients with a high ALI (median OS, 6.8 months vs. 15.8 months; *p* < 0.001), and patients with a low mALI had shorter OS than patients with a high mALI (median OS, 6.8 months vs. 16.5 months; *p* < 0.001) (Fig 1). There was no significant difference in estimates of MST between low ALI and low mALI (z = 0.000, *p* = 1.000) and between high ALI and high mALI (z = 0.330, *p* = 0.740).

Univariable Cox regression analysis revealed that both low ALI and low mALI were significantly associated with shorter OS (hazard ratio [HR], 2.10 for low ALI and 2.20 for low mALI, *p* < 0.001). Other poor prognostic factors in univariable analyses were advanced age (≥ 65 years), extensive disease, poor performance status (PS), supportive care only, and elevated LDH. Multivariable analysis showed that low ALI was an independent prognostic factor for shorter OS (HR, 1.67, *p* = 0.004), along with advanced age (HR, 1.49, *p* = 0.045), extensive disease (HR, 2.27, *p* < 0.001), supportive care only (HR, 7.86, *p* < 0.001), and elevated LDH (HR, 1.45, *p* = 0.037) (Table 3). In internal validation using a bootstrap resampling technique, low ALI (bootstrap frequency, 98.1%), advanced age (76.0%), extensive disease (100%), supportive care only (100%), and elevated LDH (76.8%) were all reliable prognostic factors. In addition, goodness of fit of this final prognostic model was not significantly increased by adding mALI as an extra variable (LR difference = 2.220, *p* = 0.136).

**Table 3:** Results of univariable and multivariable analyses of overall survival.

Variables	Univariable analysis		Multivariable analysis	
	HR (95% CI)	*p* value^a^	HR (95% CI)	*p* value
**Male sex**	1.21 (0.74–1.96)	0.446		
**Age ≥ 65 years**	1.90 (1.31–2.75)	0.001	1.49 (1.01–2.20)	0.045
**Extensive stage**	2.93 (1.99–4.32)	< 0.001	2.27 (1.52–3.40)	< 0.001
**ECOG PS ≥ 2**	2.65 (1.84–3.82)	< 0.001		
**Supportive care only**	11.74 (7.03–19.61)	< 0.001	7.86 (4.65–13.29)	< 0.001
**Charlson comorbidity Index ≥ 1**	1.33 (0.93–1.91)	0.118		
**Current/ex-smoker**	0.79 (0.46–1.38)	0.413		
**Elevated LDH (≥ 486 U/L)**	1.54 (1.10–2.17)	0.011	1.45 (1.02–2.05)	0.037
**Low ALI (ALI < 31.1)**	2.10 (1.50–2.94)	< 0.001	1.67 (1.17–2.37)	0.004

Abbreviations: HR, hazard ratio; CI, confidence interval; ECOG PS, Eastern Cooperative Oncology Group performance status; BMI, body mass index; LDH, lactate dehydrogenase; ALI, Advanced lung inflammation index

^a^Log-rank test

## Discussion

Inflammation plays key roles in immune response to pathogens and in tissue repair, but can become chronic and promote the generation of reactive oxygen and nitrogen species, and stimulate angiogenesis and cellular proliferation, which play key roles during carcinogenesis [14, 15]. Furthermore, epidemiological evidence implicates systemic inflammation in the etiology of cancer and indicates its prognostic significance in different cancers [3, 4, 16–18].

Cancer cachexia is a multi-factorial syndrome defined as an ongoing loss of skeletal muscle mass (with or without loss of fat mass) that cannot be reversed by conventional nutritional support and leads to progressive functional impairment [19]. The cachexia status is considered to be the clinical consequence of interactions between the tumor, the host metabolism, and pro-inflammatory cytokines [20]. As cancer cachexia impairs quality of life and response to therapy, the estimation of body composition is of considerable important in oncologic patients, because the quantification of skeletal muscle mass became an important diagnostic component of this syndrome and an important therapeutic target [19, 21, 22].

CT provides an objective and reproducible means of quantifying skeletal muscle mass, and single cross-sectional area of muscle at L3 is regarded as the gold standard for quantifying total-body skeletal muscle mass [23]. On the other hand, measures of BMI are obviously limited in terms of evaluating sarcopenic status, because body weights do not precisely reflect body compositions, and weight loss is obscured in patients with a large tumor mass or collected fluid, such as, pleural effusion or body edema. Accordingly, we undertook to determine whether a CT-determined L3MI-based mALI has greater prognostic value than the original BMI-based ALI. The present study shows that low mALI and low ALI are both significant prognostic indicators, but that they have similar prognostic values.

Although BMI does not provide a direct measure of body fat and skeletal muscle, it has been previously shown that a low BMI is significantly associated with a sarcopenic status [24]. Furthermore, BMI and weight loss, which are common at presentation in advanced cancer patients, has been used as a conventional diagnostic criterion for cancer cachexia traditionally.

The present study has several limitations that require consideration. First, the number of patients included was small, although the study population was homogeneous in terms of histology. Second, since optimal cut-off values have not been established for ALI or mALI, we used maximal chi-squared test determined cut-off values. Furthermore, the result that mALI has no additional value beyond original ALI should be interpreted cautiously, since it does not mean CT-determined sarcopenia has no additional value than BMI based underweight, or weight loss.

Summarizing, although CT-determined L3MI provides a precise means of quantifying skeletal muscle mass, and sarcopenia is considered an independent prognostic indicator in SCLC [24], our results show mALI, which incorporated CT-determined skeletal muscle mass has no additional value beyond ALI with respect to predicting the prognosis in SCLC. We conclude ALI, based on BMI and inflammatory markers, is a simple, clinically useful prognostic indicator in SLCL.
